# A Case of *Staphylococcus aureus* Septic Arthritis After COVID‐19 Vaccination

**DOI:** 10.1002/jgf2.70068

**Published:** 2025-09-30

**Authors:** Mineto Ohta, Rikiya Kanba, Masataka Kudo, Kazuki Kawashima, Kenji Namiki

**Affiliations:** ^1^ Department of General Medicine Osaki Citizen Hospital Osaki Miyagi Japan

**Keywords:** COVID‐19, septic arthritis, *Staphylococcus aureus*, vaccine

## Abstract

Septic arthritis is a serious medical emergency associated with high morbidity and mortality. A 49‐year‐old man with diabetes undergoing hemodialysis developed right shoulder pain and fever following his sixth dose of the COVID‐19 vaccine. Twelve days after vaccination, his fever persisted, and computed tomography imaging revealed a fluid collection around the right shoulder joint. Culture of the synovial fluid detected methicillin‐susceptible *Staphylococcus aureus* , leading to a diagnosis. Intravenous administration of ceftriaxone was effective and de‐escalated to cefazolin and then oral cefaclor. The patient was discharged without complications. Caution is warranted because unexpected complications may arise after vaccination in individuals with multiple comorbidities.

## Graphical abstract

A 49‐year‐old man with diabetes undergoing hemodialysis developed right shoulder pain and fever following his sixth dose of the COVID‐19 vaccine. The culture of the synovial fluid detected *Staphylococcus aureus* , leading to a diagnosis of septic arthritis.

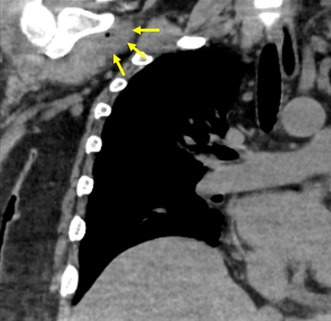

## 1 Background

Septic arthritis is an important medical emergency of acute monoarticular arthritis because failure to initiate appropriate treatment can lead to irreversible joint destruction. The incidence rate is 4–10 cases per 10,000 patients per year in European countries, and the reported fatality rate is approximately 11% [1]. Diagnosis is confirmed by detecting bacteria in synovial fluid; however, it is often made clinically through a combination of patient history and diagnostic investigations. Risk factors for septic arthritis include advanced age, skin and soft tissue infections, intravenous drug use, immunosuppression, rheumatoid arthritis or osteoarthritis, joint prostheses, alcoholism, diabetes, and prior intra‐articular corticosteroid injections. Infection could be introduced into a joint either by hematogenous spread or direct inoculation [1]. Early administration of appropriate antibiotics is important for treatment to prevent joint dysfunction. According to UK guidelines, antibiotics are typically given for up to 6 weeks; this begins with 2 weeks of intravenous treatment followed by a transition to oral treatment [2]. There have been few reported cases of septic arthritis following vaccination [3, 4, 5, 6]. In cases of septic arthritis after vaccination, it is necessary to consider iatrogenic infection in addition to the usual hematogenous infection. We herein present a case of septic arthritis following a patient's sixth dose of the COVID‐19 vaccine.

## 2 Case Presentation

A 49‐year‐old man received his sixth dose of the COVID‐19 vaccine (monovalent XBB 1.5), administered intramuscularly into the right deltoid region by a doctor following disinfection with an alcohol swab. He developed right shoulder pain 5 days after vaccination. His fever had risen to approximately 38.9°C by day 10. A blood test performed at the previous hospital revealed an elevated white blood cell (WBC) count of 14.1 × 10^9^/L and a C‐reactive protein (CRP) level of 200.0 mg/L, suggesting a bacterial infection. As a result, treatment was initiated with cefcapene pivoxil hydrochloride hydrate (200 mg/day) and acetaminophen (1800 mg/day). On day 12, his fever had decreased to 37.2°C; however, signs of inflammation persisted (WBC, 10.6 × 10^9^/L; CRP, 280.0 mg/L). Due to the ongoing symptoms, he was referred to our hospital for further evaluation and treatment.

At the initial presentation, his vital signs were as follows: body temperature, 38.7°C; blood pressure, 129/72 mmHg; pulse, 81 beats/min (regular); respiratory rate, 18 breaths/min; and oxygen saturation, 93% (room air). His right shoulder presented limited joint mobility due to severe pain. There were no visible abnormalities in the skin at the vaccination site.

His medical history included diabetes mellitus and hypertension, and he underwent hemodialysis three times a week because of diabetic nephropathy. He had a dialysis shunt in his left forearm. Additionally, he had a history of cervical sprain, right hand tendonitis, congestive heart failure, perforating dermatitis, erysipelas, and benign paroxysmal positional vertigo. There was no history of drinking alcohol. Prior to this episode, he was not receiving treatment for any inflammatory diseases and was not using immunosuppressants. There was no history of allergic reactions to vaccinations.

**FIGURE 1:**
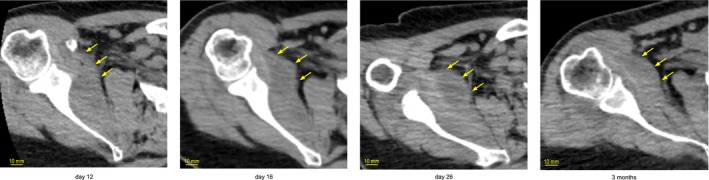
Plain computed tomography images of the abscess around the right shoulder joint. Axial computed tomography images (5‐mm slices) were taken at 12 days (admission), 16 days, 26 days, and 3 months. At the time of admission, the abscess measured 50.8 × 23.8 × 40.8 mm and appeared as a low‐density fluid collection with air inside. After 3 weeks of treatment, the abscess had decreased in size to 35.3 × 19.3 × 27.5 mm and had become organized.

The complete blood count and biochemistry data at the time of the first visit are shown in Table 1. CT imaging revealed fluid accumulation around the right shoulder joint (Figure 1). Based on his symptoms and CT findings, septic arthritis was suspected. An orthopedic surgeon performed an exploratory puncture around the right shoulder joint, which yielded a small amount of viscous, purulent synovial fluid. Gram‐positive cocci were detected. The patient was diagnosed with septic arthritis and admitted to the general medicine department for comprehensive management. Considering the possibility of sepsis with anuric patient, treatment was initiated with 2 g intravenous ceftriaxone every 24 h, administered after dialysis. Following admission, the patient's right shoulder pain and inflammatory response gradually improved. A CT scan performed on day 16 showed persistent effusion in the right subscapularis muscle. Because drainage of the abscess was deemed challenging, conservative antibiotic treatment was continued. On day 17, *Staphylococcus aureus* was detected in the synovial fluid culture, while blood cultures remained negative. The culture results showed that *S. aureus* was sensitive to all tested antibiotics; accordingly, the antibiotic was switched to 2 g intravenous cefazolin every 24 h. By day 39, the CRP level had decreased to 23.2 mg/L, and the antibiotic was changed to oral cefaclor (500 mg/day). Over time, the abscess became organized (Figure 1). As the pain improved, the restriction of shoulder joint movement disappeared, and the patient was discharged on hospitalization day 45 without any complications.

**TABLE 1:** Laboratory data at the time of hospitalization.

Parameter	Reference range (adults)	Patient's result
White blood cells (×10^9^/L)	4.5–11	10.7
Differential white blood cell count		
Neutrophils (×10^9^/L)	2.6–8.5	8.9
Lymphocytes (×10^9^/L)	0.77–4.5	0.9
Monocytes (×10^9^/L)	0.14–1.3	0.6
Basophils (×10^9^/L)	0–0.55	0.22
Eosinophils (×10^9^/L)	0–0.22	0.01
Hemoglobin (g/L)	100–120	101
Platelets (×10^9^/L)	150–350	238
Total bilirubin (μmol/L)	5.1–20.5	6.3
Aspartate aminotransferase (U/L)	5–25	29
Alanine aminotransferase (U/L)	3–30	34
Lactate dehydrogenase (U/L)	124–222	110
Uric acid (mmol/L)	0.15–0.47	0.71
Blood urea nitrogen (mmol/L)	2.9–7.1	12.3
Creatinine (μmol/L)	80–110	598
Estimated glomerular filtration rate (mL/min/1.73 m^2^)	> 90	7.8
Total protein (g/L)	67–83	72
Albumin (g/L)	35–54	26
Creatine kinase (U/L)	30–170	66
Hemoglobin A1c (%)	4.7–6.2	10.2
C‐reactive protein (mg/L)	< 8.0	356.0
B‐D glucan (pg/mL)	0–11	< 11.0
Brain natriuretic peptide (ng/L)	< 100	171.9
Rheumatoid factor (kIU/L)	0–15	12
Anti‐cyclic citrullinated peptide antibody (U/mL)	0–4.5	0.6
Synovial fluid		
Urate crystals	Not detected	Not detected
Calcium pyrophosphate crystals	Not detected	Not detected

## 3 Discussion

Receiving multiple doses of COVID‐19 vaccines was proven effective during the Omicron period, and vaccination is recommended for all eligible individuals [7]. This patient had multiple comorbidities, including diabetes mellitus, obesity, cardiovascular disease, and end‐stage renal disease. It is well established that patients with end‐stage renal failure exhibit a less robust and less durable antibody response despite achieving seroconversion following vaccination. Therefore, a third or booster dose is necessary to generate an optimal antibody response [8]. Furthermore, structured clinical follow‐up is recommended for all vaccinated patients with heart failure [9].

There have been few reported cases of septic arthritis following vaccination, including COVID‐19 vaccination [3, 4, 5, 6]. The underlying cause may be related to the vaccine itself, the vaccination procedure, or simply an incidental occurrence; no definitive cause has been described. Recommended precautions include the use of alcohol‐based disinfectants with aseptic techniques, avoiding injections in the anterior and upper third of the deltoid muscle, and using patient‐specific needles [10]. In this case, the route of infection—whether hematogenous spread or direct inoculation—remains uncertain because there was no redness or swelling in the right deltoid area, and blood cultures were negative. Blood count and biochemistry tests of the synovial fluid were not performed and should have been performed for differential diagnosis. The association between the events and vaccination is considered ‘possible’ according to World Health Organization–Uppsala Monitoring Centre (WHO‐UMC). On admission, the patient was unable to lift his arm. Early diagnosis and appropriate treatment led to a favorable outcome, with the patient regaining the ability to elevate his arm. It is important to closely monitor the progress of individuals with multiple comorbidities following vaccination.

## Author Contributions

M.O. and R.K. conceptualized the study. M.O. conducted the investigation and prepared the original draft. M.K. supervised the work. K.N. managed the project. All authors have reviewed and approved the final version of the manuscript.

## Ethics Statement

Ethical review and approval were waived for this case report because it does not constitute a research study.

## Consent

Informed consent was obtained from the patient for the publication of this case report and any accompanying images. Written consent was secured for the publication of this paper.

## Conflicts of Interest

The authors declare no conflicts of interest.

## Data Availability

The data that support the findings of this study are available from the corresponding author upon reasonable request.
